# Microplastic Particles and Fibers in Seasonal Ice of the Northern Baltic Sea

**DOI:** 10.3390/toxics12080542

**Published:** 2024-07-26

**Authors:** Janika Reineccius, Mareike Heck, Joanna J. Waniek

**Affiliations:** 1 Leibniz Institute for Baltic Sea Research, Warnemünde, Seestraße 15, 18119 Rostock, Germany; 2 Institute of Biosciences, University of Rostock, Albert-Einstein-Straße 3, 18059 Rostock, Germany

**Keywords:** microplastic, microfibers, seasonal sea ice, ice core, Baltic Sea, Gulf of Bothnia

## Abstract

Microplastic pollution is a pervasive issue, with remarkably high concentrations observed even in the most remote locations such as Arctic sea ice and snow. The reason for such large microplastic abundances in sea ice is still speculative and applies mainly to saline or freshwater conditions. In this study, we investigated seasonal ice core samples collected in March 2021 from the northern Baltic Sea (Gulf of Bothnia) for their microplastic distributions. The Baltic Sea is characterized by low salinity and can be ice-covered for up to six months annually. Microplastics were analyzed in the melted ice samples using an adsorption technique and Raman microscopy to identify their abundances, colors, shapes, and sizes to calculate their masses. Due to the strong dynamic of the ice layer and the repeated melting and freezing processes during the ice formation, no discernible trends in microplastic abundances, masses, or polymer types were observed throughout the ice core length. The average microplastic abundance (±SD) in the Baltic Sea ice was determined to be 22.3 ± 8.6 N L^−1^, with 64.9% of the particles exhibiting a particulate shape and 35.1% having a fibrous shape. The most prevalent polymer type was polyethylene terephthalate (PET), accounting for 44.4% of all polymers. This is likely due to the high proportion of PET fibers (93.8%). The majority of particle-shaped microplastics were identified as polyethylene (PE; 37.2%), followed by PET (17.2%), polyvinyl chloride (PVC; 15.9%), and polypropylene (PP; 15.9%). No correlations were found between microplastic concentrations and proximity to land, cities, industries, or rivers, except for PP mass concentrations and particle sizes, which correlated with distances to industries in Luleå, Sweden.

## 1. Introduction

Plastic has attained a high status in contemporary society, facilitating significant advancements in medicine, public health [1], hygiene, and the efficiency of everyday life. Its durability, lightweight nature, and versatility in applications have led to an exponential increase in global production, despite the lack of a suitable infrastructure for managing such large plastic amounts. According to estimates, by 2015, the total mass of plastic waste globally had reached 6300 million metric tons (MT) [2], a figure that has continued to rise [3]. As a result of inadequate handling and poor waste management, it is estimated that 4.8–12.7 million MT of plastic is discharged into the ocean each year [4].

The accumulation of large quantities of plastic debris in the marine environment has resulted in several adverse consequences for ecosystems, animals, and even humans [5]. In particular, the presence of small plastic items, such as microplastics (MPs), defined in the size range between 1 and 5000 μm, has been widely documented in marine habitats and biota around the globe [6]. The detrimental effects of MPs on marine animals, which ingest MPs through their food, are diverse and pervasive. In seabirds and sea turtles, microplastics have been shown to reduce feeding activity [7], while marine isopods also exhibit decreased feeding behavior [8]. Marine mussels exposed to MPs show altered immune responses [9], and sea cucumbers and lugworms experience oxidative stress [10,11]. Additionally, MPs negatively impact reproductive performance in various marine species, including crabs [12] and oysters [13], as well as smaller marine organisms [14,15,16].

As a result of the lipophilic properties of MPs, the toxicological effects can be further exacerbated by their ability to absorb toxic chemicals from their surroundings, including persistent organic pollutants (POPs) [17,18,19] or heavy metals [20,21]. Furthermore, toxic contaminants added during the manufacturing process may leach out, such as monomer residues or additives like phthalates, bisphenol A, or organophosphorus esters [22,23,24]. The toxicity of MPs, including sorption capacities and leaching extent of pollutants, can be further associated with the shape, size, and polymer type [25]. An extended surface-to-volume fraction caused by a porous structure or fragmented surfaces provides increasing sorption and leaching areas. The shape of MPs even influences their toxicity by affecting the retention times of MPs in animal intestines, as demonstrated for the zebrafish by Qiao et al. [26].

MPs exhibit various characteristics that influence their distribution and retention time in marine environments, which change with weathering [27]. The retention time, in turn, affects their bioavailability and the threat they pose to marine wildlife [28]. External physical factors such as currents, gyres, river discharges, and weather, along with biological processes like bio–fouling and aggregation, as well as human activities, further influence MP distribution [29,30,31]. Due to the multitude of influencing factors, tracing or predicting the ultimate fate of MPs is challenging. Significant knowledge gaps remain in understanding MP behavior in marine systems, particularly in remote locations or regions difficult to access by ship, such as areas covered by sea ice. Up to 34 million km^2^ of the global oceans are seasonally covered by sea ice in the Arctic and Antarctica alone [32], which is about 9% of the total global ocean area with 361 million km^2^ [33]. This vast expanse of sea ice represents one of the largest biomes on Earth, playing a crucial role in the survival of marine organisms [34]. In 2014, Obbard et al. [35] discovered and published the presence of MPs in Arctic sea ice for the first time. Their findings indicated that the concentrations of MPs in sea ice (38 to 234 MPs m^−3^) were more than two orders of magnitude higher than previously reported for the North Pacific Subtropical Gyre (0.34 and 0.12 MPs m^−3^), which is considered the most polluted oceanic gyre globally [36]. Following this discovery, several MP studies were conducted in the Arctic, including studies of fast ice, seasonal sea ice, and even snow, pond water, and surrounding waters in the Arctic [37,38,39,40,41], aiming to understand the remarkably high MP concentrations observed in sea ice. This phenomenon of MP accumulation in sea ice was not limited to the Arctic; it was also observed in Antarctica [42], Greenland [43], the Sea of Japan [44], and Svalbard waters [45]. Sea ice has been identified as an important temporal sink for MP in the ocean, entrapping MP from the water and atmosphere [40]. For seasonal ice, this results in an annual cycle where MPs are catched in the sea ice during winter and then released into the water column as the ice melts in spring [39,46]. Nevertheless, the precise mechanisms underlying the accumulation of MPs in sea ice remain poorly understood. The freezing and accumulation processes in saline seawater exhibit notable differences from those suggested in freshwater or brackish water [47]. In saline waters, ice formation creates large brine channels, increasing ice porosity and facilitating the transportation of substances, including MPs, within the ice [47,48]. This is not the case for freshwater, where ice is expected to freeze in well-stratified layers with MPs concentrated at the surface [47].

**Figure 1:**
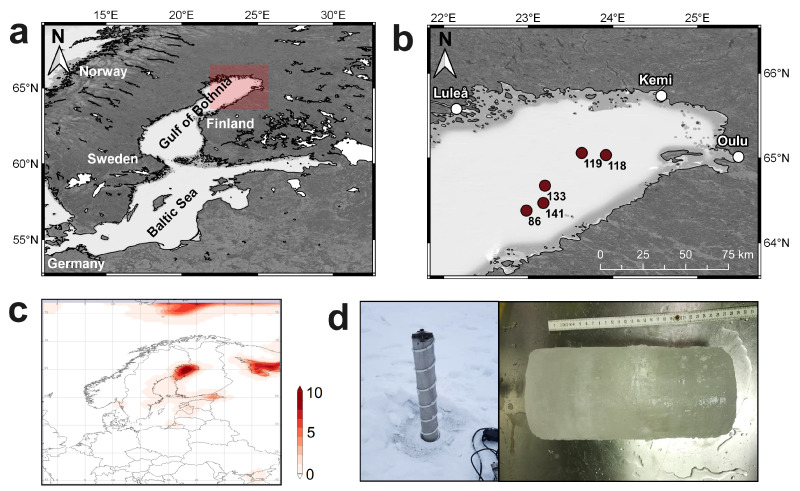
Overview of the sampling site (**a**) and respective sampling stations (**b**) for ice coring in the Gulf of Bothnia (Bothnian Bay) as the northernmost part of the Baltic Sea. Below, the average sea ice covered fraction for the sampling month March 2021 (provided by Global Modeling and Assimilation Office (GMAO) (2015), MERRA-2 tavgU_2d_ocn_Nx: 2d,diurnal, Time-Averaged, Single-Level, Assimilation, Ocean Surface Diagnostics V5.12.4, Greenbelt, MD, USA, Goddard Earth Sciences Data and Information Services Center (GES DISC), Accessed: [19.04.2024], 10.5067/KLNAVGAX7J66) is illustrated (**c**). Photographs of the ice coring procedure and a final ice core are illustrated in (**d**) (photographs by S. Papenmeier, IOW).

To the best of our knowledge, the processes occurring in brackish water have only been investigated by Geilfus et al. [49]. They examined sea ice cores from the Swedish and Finnish shorelines of the Gulf of Bothnia (Baltic Sea) adjacent to potential riverine sources. Their study found that the MP concentrations in these sea ice cores were not significantly higher than those in surface waters [49]. This finding contrasts with studies conducted in saline waters [41] and freshwater investigations [50]. To elucidate these discrepancies, this study examines sea ice cores collected from the central Gulf of Bothnia, at distances >29 km from the coast (Figure 1a). Sampling close to river inflows would primarily reflect the pollution situation of the rivers themselves rather than the broader Baltic Sea. By sampling further from potential riverine sources of MPs, we aim to gain a more comprehensive understanding of the pollution situation in the entire area. This study aims to investigate the processes related to MP accumulation and distribution in brackish sea ice cores. To achieve this objective, five sea ice cores were analyzed for their MP abundances, masses, sizes, and polymer types. By examining these parameters, we aim to provide insights into the MP pollution in the brackish waters of the Gulf of Bothnia and to contribute to a better understanding of MP behavior in such environments.

## 2. Materials and Methods

### 2.1. Sampling Site

The Baltic Sea (53°–66° N, 10°–30° E), with an average depth of 55 m, is an intracontinental marginal sea of the Atlantic Ocean in northern Europe and is the largest brackish sea on Earth. It is characterized by microtides, which reach a maximum height of 23 cm [51] and exert a low influence on the surrounding area, comparable to that of waves [52]. Due to its geographical location and extensive north-south extent, the Baltic Sea encompasses two distinct climate zones: the humid temperate (oceanic) and the continental climate zone. Consequently, the temperature of the Baltic Sea varies considerably depending on the location, season, and depth. During winter months, average water temperatures can drop down to 1.7 °C in March, while in summer, temperatures are around 16.8 °C [53]. The prevailing cold climate conditions in the northern Baltic Sea with 0–2 °C even facilitate the sea ice formation [54]. In contrast to the Arctic, the Baltic Sea is only temporarily ice-covered. The thaw–melt cycle can also be repeated several times during a single winter. The northernmost parts of the Baltic Sea, including the Gulf of Bothnia, the Gulf of Finland, and the Gulf of Riga, can be ice-covered for up to six months each year [48], whereas the southern parts are rarely covered with ice. The brackish nature of the Baltic Sea is caused by intermittent inflows of saline water via the Danish Straits from the North Sea and large freshwater runoff by surrounding rivers and streams. This single and tight connection to saline water in the southwest results in a salinity gradient in the Baltic Sea, with saltier waters in the southwest (10–20) and nearly freshwater conditions in the north (Table 1). Consequently, the ice formation in the northern Baltic Sea, including the Gulf of Bothnia, is also similar to freshwater [48]. The surface currents in the Baltic Sea are mainly driven by wind and thermohaline effects. Wind in this area however, is strongly variable in their strength and direction. The long-term circulation shows a cyclic structure in the northermost Bothnian Bay, with northward currents along the Finish coast and southward along the Swedish coast [55].

### 2.2. Sampling Procedure

Five sea ice cores were sampled from the seasonal ice layer of the northern Baltic Sea in the Gulf of Bothnia (Figure 1) on board the research vessel Maria S. Merian (MSM) in March 2021 (Table 1) during the research expedition MSM99 (research mission “Deep Baltic”). The sampling dates and conditions are given in Table 1. The ice cores were collected by removing the upper snow layer and drilling a 12 cm diameter stainless steel corer into the ice (Figure 1c). The obtained stable ice cores were 16–20 cm in length (Table 1), which were immediately transferred to the onboard laboratory for slicing with a bone saw into 3–5 cm slices. The lowermost layers of the ice cores were discarded from further analysis due to their excessive porosity and incomplete freezing, making them unsuitable for further MP analysis. To prevent contamination, all equipment used to handle the cores was washed thoroughly with MilliQ. Nitrile gloves, rinsed with MQ prior to core handling, were worn throughout sample processing. Furthermore, any adhering particles on the ice core surface were washed with MilliQ. Core slicing was conducted under a previously cleaned fume hood, and the slices were transferred into silicone bags immediately. The core slices were stored in the freezer and remained frozen until they reached the home laboratory for further analysis.

### 2.3. Microplastic Extraction

For MP separation from the sea ice cores, slices were thawed at room temperature for 24 h into a previously rinsed glass beaker using a glass funnel covered with aluminum foil. The resulting melt water with 260–520 mL volume was then passed through a coated syringe cascade. This method was validated for seven of the most commonly used and produced plastic polymers (PE, polyethylene; PP, polypropylene; PS, polystyrene; PVC, polyvinyl chloride; PET, polyethylene terephthalate; PA, polyamide; PMMA, polymethyl methacrylate) within the size range between 182.6–1252.8 μm [56]. The recovery rates ranging between 88.7 ± 8.6% for PA and 100.0 ± 0.0% for PE, PP and PS [56]. In this method, MP particles were trapped into the inner syringe (30 mL, polypropylene, Terumo, Japan) walls, which were coated with lubricant oil (Ernst GmbH & Co. KG, Kamen, Germany) to create a lipophilic film that retains the lipophilic MP particles while non-lipophilicmaterials pass the syringes and can be removed. The melt water was passed through the syringe cascade three times to ensure a high performance. Following the MilliQ purification step to remove any residual materials, the MP particles were detached by thoroughly rinsing the syringes with a 50 °C warm, prefiltered detergent solution (glass microfiber filter Whatman™; 47 mm diameter; 0.7 μm pore size; CatNo. 1825–047).

The resulting detergent solution containing the MP particles was then filtered onto a polycarbonate membrane filter with a nominal pore size of 0.4 μm (Whatman Nuclepore Track-Etched Membranes WHA110637, diameter 25 μm). The filtration funnel was rinsed thoroughly with MillQ to remove detergent residues, followed by ethanol (30%, Merck KGaA, Taufkirchen, Germany) to detach particles adhering to the inner filtration funnel. Subsequently, the filter was transferred to a glass slide and covered with a coverslip to prevent potential airborne contamination.

### 2.4. Blank Tests

Parallel blanks were run for each ice core sample to simulate potential contamination during the sample processing in the laboratory. As core slices were sliced under a clean fume hood, rinsed with Milli-Q water, and transported in plastic-free silicone bags, no MP contamination was expected to occur in these first steps. However, after thawing, the contamination risk increased. The thawing process was artificially created by filling 400 mL of MilliQ into a funnel and a glass beaker as the samples were passing during the melting process. After 24 h (melting time of the samples), the blank water was further processed the same way as the samples.

### 2.5. Microplastic Identification with Raman Microscopy

For MP identification, the Raman spectrometer LabRAM HR 800 Horiba Jobin YVON, Darmstadt, Germany, which is equipped with an integrated Olympus BX41 microscope with variable lenses and camera, was employed. All filters were manually scanned for potential MP particles under transmitted light at an objective magnification of 10× (window size 0.7 × 0.9 mm). Only those particles that were unambiguously not MP based on their similar surface structure, shape, and color to particles previously identified as negative for MP on the same filter were not included in potential MP particles. Suspected MPs were measured at 50× magnification with a red laser (633 nm, 17 mW, air-cooled HeNe laser) at 10 s integration time and 10 accumulation scans in the wavenumber range 50–3800 cm^−1^. The minimum measurable particle size with this method was 10 μm. Following measurement, each particle was photographed at either 10× or 50× magnification, dependent on MP size, for subsequent size and mass calculations. Particle spectra were analyzed using the open-source software Open Specy 1.0 (www.openspecy.org; [57]) database. Only clear spectra with a matching degree >80% with the polymer database were assumed to be MP. The resulting MP items were then subtracted from the MP counts and masses for each ice core slice sample.

### 2.6. Image Analysis and Calculations

The microscopic images of MP items were applied to calculate their volume by using the open-source software FIJI-ImageJ 1.53o [58]. The particle volume was measured using the pixel brightness [59] optimized for each image to ensure maximum accuracy, or the particle volume was measured manually if the threshold was not usable. Particle volumes were calculated according to the method by Kumar et al. [60] and Poulain et al. [61]. In this method, the two-dimensional (2D) particles were assumed to be ellipsoids, for which the maximum axis (Ferret diameter) and the minimum axis were used to calculate the volume as described in Equation (1):

(1) $$V_{P}=\pi \times \frac{l_{P}\times w_{P}\times w_{P}}{6}$$

where the particle length (*l_P_*) is the maximum measured axis (Ferret diameter) and the particle width (*w_P_*) is the minimum axis of the photographed particle. The volumes of fibers or elongated-shaped MPs were calculated using the general formulae for cylindrical bodies (Equation (2)):

(2) $$V_{F}=\pi \times {\left(\frac{w_{F}}{2}\right)}^{2}\times l_{F}$$

The fiber width (*w_F_*) was measured at five distinct locations along the fiber. The average of these widths was employed to calculate their volume. (*l_F_*) represents the fiber length. To differentiate between fibers and elongated particles, a threshold of <5% was established for the width-to-length fraction of fibers. Elongated MPs were defined as having a width-to-length fraction of ≥5%, which were included in the particle-shaped MPs in the results.

The resulting volumes were multiplied by the respective polymer densities for each MP, thereby obtaining the MP mass. For this calculation, the polymer densities published by Hidalgo-Ruz et al. [28] were utilized. In the present work, the resulting MP mass concentrations were expressed in μg L^−1^, and the MP abundances were given in N L^−1^. Both terms were determined for all five collected ice core samples, while two of these cores were analyzed with a full vertical resolution throughout the ice core length in 3–5 cm steps.

### 2.7. Statistical Analyses

A Neyman–Pearson correlation test was conducted between two data sets. Multiple correlation analyses were performed using the open-source statistic program Past (version 4.02) or Python software (Spyder version 5.2.2). Correlation tests were conducted to investigate potential associations between MP abundances, masses, or polymer types identified in ice core samples and distances to specific ambient locations (industries, rivers, land, cities), a north–south or east–west gradient, or ice core length. The specific locations were selected based on their potential to serve as sources of MP. MP is of anthropogenic origin, with potential sources generally located in adjacent coasts (Sweden and Finland), large cities with high population densities (Oulu), and rivers traversing numerous urban and industrial areas (with the largest concentrations in Raahe and Luleå). The distances utilized for correlation testing are between 47 and 170 km to the industries, cities, and Kemi River, while 29–78 km are between the stations and adjacent land. More details are provided in the Supplemental Material (Table S1). To identify potential differences in MP occurrences in the surface layer compared to the residual ice core, a *t*-test (equal means) and Mann–Whitney U (equal medians) were performed. For all tests, a confidence interval of 95% was applied.

## 3. Results

### 3.1. Microplastic in the Northern Baltic Sea

**Figure 2:**
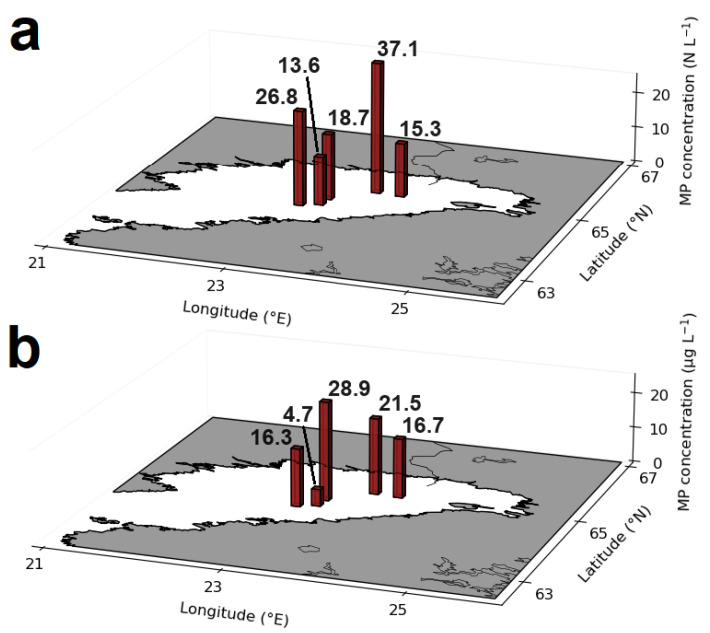
Total MP abundances (**a**) and MP masses (**b**) detected in ice core samples of the northern Baltic Sea. Numbers displayed at the bars represent the individual concentrations.

The presence of MPs was confirmed in all five ice core samples from the Gulf of Bothnia, with a total number of 209 particles. A total of four MP particles were found in all five blank samples, with fewer than one MP item per blank sample. The mean blank-corrected MP abundance and mass found per ice core sample were 22.3 ± 8.7 N L^−1^ and 17.4 ± 7.6 μg L^−1^, respectively (Supplemental Material Table S2). The particulate shape (fragments, sheets, elongated particles) was the predominant MP fraction, accounting for 64.9%, while the fibrous shape accounted for 35.1%. No spherical MP particles were identified in any of the samples. The highest MP abundance was observed at the northernmost station (119), with 37.1 ± 9.0 N L^−1^. The southernmost station (86) exhibited the second-highest MP abundance, with 26.8 ± 31.3 N L^−1^ (Figure 2). The lowest MP abundance and mass were detected at station 141, with 13.8 ± 6.5 N L^−1^ and 4.7 ± 2.0 μg L^−1^, respectively. The higher MP mass but lower MP abundance detected for station 133 compared to the other ice core stations can be attributed to the larger MP sizes at station 133.

**Figure 3:**
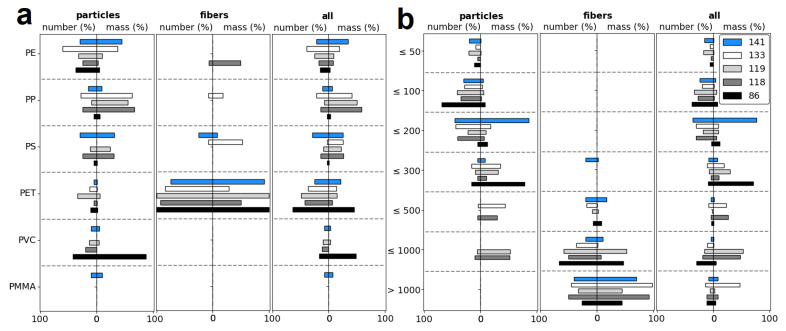
Polymer fractions (**a**) and size fractions (in μm) (**b**) of the detected MPs in the ice core samples for particles, fibers, and all MPs as a sum. Fractions were calculated for the MP numbers and masses, respectively.

The mean MP size was 137.6 ± 133.0 μm for the particulate-shaped MPs, while fibers were significantly longer with 2423.1 ± 3758.0 μm length. MP particles are most prevalent in the size class 50–100 μm (38.5%), with 95.2% being below 300 μm (Figure 3b). In contrast, the fiber length is primarily longer than 300 μm (98.8%). The longest fibers were found at the southernmost station (3490.5 ± 5751.8 μm), where the fiber abundance was also high. The MP particle sizes at the same station were much smaller and were dominant in the size class 50–100 μm. The smallest fiber sizes were observed at the northernmost stations, 118 and 119, suggesting a potential correlation between the fiber size and latitude, but the correlation coefficient was weak (|r| = 0.80; *p* = 0.10). In contrast, the MP particle size exhibits a negative correlation with the distance to two major industrial sites in Raahe (|r| = 0.89; *p* = 0.04) and Luleå (|r| = 0.90; *p* = 0.04). Nevertheless, no correlation was observed between the abundance or mass of MP particles or fibres and distances to industries, rivers, adjacent land, or latitude.

The ice core sample exhibited a prevalence of PET at 44.4%, followed by PE (23.9%), PP (10.7%), PS (10.2%), PVC (9.8%) and PMMA (1.0%). The substantial presence of PET can be attributed to the fiber proportion, which comprises 93.8% PET (Figure 3a). MP particles only contribute 17.2% to PET, with the most frequent polymer type being PE, with 37.2%. The highest PET content was detected at station 86, with 18.9 ± 24.4 N(PET) L^−1^ and 10.5 ± 8.6 μg L^−1^. Concentrations of PVC concentrations were also the highest at the same station (Figure 3a). The highest PE abundances and masses were found at station 119, with 9.1 ± 5.8 N(PE) L^−1^ and 2.0 ± 2.2 μg L^−1^, respectively.

No correlation was found between the polymer types and the adjacent industries, land masses, or rivers, with the exception of PP. A strong correlation was found between PP masses and the distance to Luleå (|r| = 0.90; *p* = 0.04), indicating that higher PP masses are associated with lower distances. This is also the case for Raahe, although the correlation with PP is weaker (|r| = 0.84; *p* = 0.08).

### 3.2. Microplastic Distribution through the Ice Core Length

**Figure 4:**
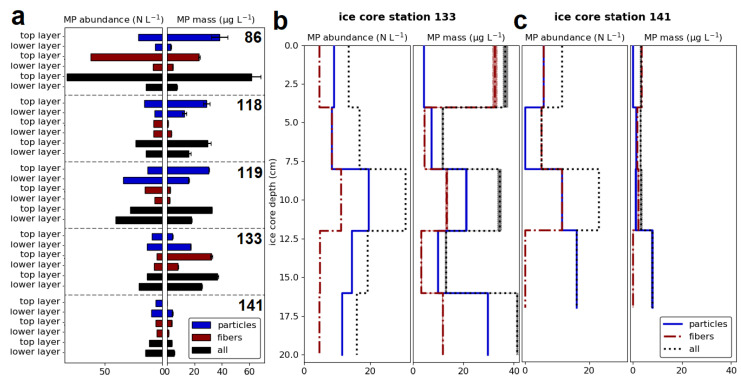
MP distribution through the ice core length of all five ice core samples (**a**) for the top ice layer (0–5 cm) and the remaining lower layers with depths depending on the ice thickness and core properties. All abundances and masses were given for particles (blue), fibers (red), and all (black) detected MPs as a sum. The depth profiles illustrate the MP distribution through the entire ice core for stations 133 (**b**) and 141 (**c**). The shaded areas surrounding the MP mass lines in (**b**) and (**c**) represent the small mass ranges resulting from the use of polymer-specific densities for mass calculations (density ranges for each polymer type).

Each ice core underwent a comparative analysis of MP concentrations observed at the top core layer and the lower layers. Two of the five ice cores were additionally subjected to depth-resolved analysis over the entire core length in 3–5 cm increments. For the southernmost ice core (station 86), a clear distinction was observed between the top and lower layers (Figure 4a). The top core layer exhibited significantly higher MP abundances and masses (abundance: t = −8.51, *p* < 0.01; mass: t = −20.92, *p* < 0.01). Such a clear pattern was not observed for the other ice cores, and statistical analysis did not reveal any significant differences. For one of the five cores (station 119), the MP particle abundance was even higher in the lower layers than in the top layer (t = −4.49, *p* = 0.03). Upon examination of the fiber masses alone, it becomes evident that the fiber abundances of the three southern stations (86, 133, 141) are higher in the top layer than in the lower layers, with a statistically significant difference for stations 86 (t = −10.10, *p* < 0.01) and 133 (t = −9.61, *p* < 0.01).

The review of the entire ice core length of stations 133 and 141 revealed no explicit vertical trend (Figure 4b,c). As discovered for station 133, the top layer exhibits the highest MP masses, reasoned by the high fiber mass content. Because the fiber abundance at the top layer is comparatively low compared to the mass, the fibers at the top layer are large. Furthermore, the middle (8–12 cm depth) and the deepest part of the ice core exhibited high MP particle mass fractions, primarily attributable to MP particles. Given the high MP particle masses in the deepest core layer relative to the particle abundance, MP particles are notably large in size in this core depth. The highest fiber masses at the top ice core layer were also observed for station 141, as well as the highest particle masses in the deepest core layer.

## 4. Discussion

### 4.1. Microplastic Distribution in the Gulf of Bothnia

The interpretation of MP abundances and masses occurring in the marine environment is complex, as potential sources are ubiquitous, and MPs behave differently depending on the external conditions. These conditions include water salinity, currents, winds, and temperature, as well as particulate properties such as shape, polymer density, and even their weathering state [27]. The primary sources of MPs in the marine system have been identified as rivers and the atmosphere; however, in the case of the Baltic Sea, numerous rivers warrant consideration as potential sources of MPs. Atmospheric inputs are primarily sourced from adjacent cities and industrial areas, and the land surrounding the Gulf of Bothnia especially in the north (Bothnian Bay) is close at each point (<80 km). Consequently, tracing the origin of detected MP abundances and masses is challenging, even when the sample size is increased. Nevertheless, we identified some correlations that suggest potential sources and processes influencing the MP distribution within the Gulf of Bothnia. Through correlation tests with multiple potential MP sources, we observed that MP particle sizes increased with decreasing distances to Luleå and Raahe. Luleå is the 25th largest city in Sweden and borders the Gulf of Bothnia in close proximity to the sampling stations. The city of Luleå is home to two iron and steel industries situated near the water and the river Luleälven, which flows through the city. This combination of industries and the nearby river provides a high capacity for transporting anthropogenic particles from Luleå and previously passed cities to the sea. Although Raahe is not situated on a major river, it is home to the largest steel and iron factory in the surrounding area. Therefore, MP particles might be introduced at those cities to the Gulf of Bothnia, leading to larger MP particles with smaller distances to those cities. With increasing distances to those potential MP sources, MP particles are significantly smaller, potentially caused by sinking or fragmentation.

In addition to the observed correlation between MP particle sizes and distance to Luleå, a similar correlation is observed for PP masses, which further supports the hypothesis that the Swedish coast city is a potential MP source for the Baltic Sea. However, this correlation only explains half of the observations regarding MP abundances and masses, as the general concentrations are not significantly different from the distances to industries, cities, rivers, or latitudes. In contrast, a negative correlation was observed for fiber sizes, with decreasing fiber lengths associated with decreasing distance to Raahe. The sources of fibers and other small MP particles may differ from those of larger MP particles. The northward current passing the Finnish coastline may be responsible for transporting MPs from more southern regions to the northern Gulf of Bothnia. Potentially, this source has a higher fiber content due to the higher population densities close to the coastlines in the southern parts of the Baltic Sea. Previous studies have demonstrated that large cities are the primary source of MP fibers [62,63]. This is due to the fact that a large population results in a high demand for textiles, as well as a high number of washing machines, which produce wastewater that is not adequately purified in wastewater treatment plants regarding MPs [64]. Fibers can be introduced to the Baltic Sea via rivers or directly via the city dust [65]. Long fibers possess a large surface-to-volume ratio, similar to smaller particles, and therefore exhibit greater surface tension, which impedes the sinking of fibers into deeper water layers. Consequently, fibers can further be transported via winds or surface currents, such as the northward current in the Gulf of Bothnia, and reach the sampling stations. This would be one potential explanation for the larger fiber sizes observed in the south. However, the observed local differences may also be attributed to the ice formation processes occurring in the Baltic Sea, as these can vary significantly depending on the sampling station [66].

### 4.2. Ice Formation Processes in the Gulf of Bothnia

The seasonal ice formation in the Baltic Sea is characterized by significant dynamic processes. Within the same winter, the surface water undergoes multiple freeze–thaw cycles [47]. It is generally understood that sea ice accumulates larger MP numbers compared to the surrounding seawater [39,41,50]. A steady input to the surface ice layer occurs via atmospheric fallout. In addition, the strong stratification caused by the sea ice layer can also be responsible for further MP accumulation in the ice, as surface water dynamics provoked by winds and waves are strongly reduced. In the calmed and covered water, light MPs that were mixed through the upper water layers before ice-building processes can rise to the ice–water boundary to be trapped by the ongoing ice formation.

In the sampling year 2021, the ice formation dynamics in the Baltic Sea were observed to undergo several freeze–thaw cycles, as well as rafting and ridging within the sampling zones [66]. The first ice formation was observed for stations 118 and 119 on 28 January 2021, with a thickness of 15–30 cm. One week later, ice was also observed at the other three stations. On February 12, the ice layer underwent a reduction in thickness due to melting, but it subsequently grew again over the following two weeks and remained frozen until the sampling dates in March. Throughout this period, the ice sheet exhibited a strong tendency to ridging and rafting, resulting in a significant mixing of the ice layers.

The low salinity of the Baltic Sea in the Gulf of Bothnia is conducive to ice formation processes that are similar to those observed in freshwater lakes with a negligible brine content within the ice and the highest MP concentration at the top and bottom ice layers [47]. However, due to the influence of ridging and rafting processes, the previous top ice layer can be displaced to the ice bottom or twisted in other directions. The ice at stations 118 and 119 was the oldest and may have had the most time to accumulate MPs. It reached its maximum thickness (30–50 cm) on February 26, equal to stations 133 and 86. However, the ice thickness of station 141 decreased at the same date to a thickness of 5–15 cm. This thawing process may be the reason for the lower MP concentrations at station 141 (Figure 2). Trapped MP particles were released, and accumulation started again but was delayed compared to the other ice core stations.

Overall, no vertical trend was observed for these five ice cores, which is consistent with the findings of Geilfuß et al. [49] conducted in the Baltic Sea and previous studies implemented in the Arctic [39,41]. In contrast to the hypothesis that MPs are redistributed via brine channels in salty waters, the ridging and rafting processes, as well as the numerous freezing and thawing cycles, are more likely to be the cause of the vertical mixing of MP concentrations in the northern Baltic Sea ice cores.

### 4.3. Comparison with Previous Studies

Most sea ice studies for MP detection have been conducted in the Arctic until now (Table 2). The first study investigating MP in ice was carried out by Obbard et al. [35] in the Arctic, where an MP abundance of (3.8–23.4) × 10^−2^ N L^−1^ was detected. In 2014, the Arctic ice was identified for the first time as an MP accumulation zone. Higher MP concentrations in the Arctic were detected by Peeken et al. [37] in pack ice and landfast ice, with abundances of 1.1–12 × 10^3^ N L^−1^. Further studies conducted in the Arctic have reported MP abundances ranging between 2–43 N L^−1^ [39,40,41]. These findings are consistent with our results, which exhibited similar MP abundances in the same order of magnitude (13.8–37.1 N L^−1^). Furthermore, the results align well with those of a previous ice core study conducted in the northern Baltic Sea (Gulf of Bothnia), with MP abundances ranging from 8 to 41 N L^−1^ [49]. Even ice cores collected in Antarctica exhibited comparable MP abundances, with 11.7 N L^−1^ [42]. The highest MP abundances in sea ice were observed in the seasonal ice cores of Novik Bay (Sea of Japan), with 428 N L^−1^ [44].

Despite the considerable distances between the Arctic, Antarctica, the Baltic Sea, and the Sea of Japan, the concentrations of MP in ice cores from these regions exhibit a high degree of consistency. It is anticipated that remote locations such as the Arctic and Antarctica will exhibit considerably lower levels of MP pollution. However, considering the seasonal nature of the Baltic Sea ice and the Sea of Japan, resulting in shorter accumulation times, it can be postulated that the abundances of MP are likely to be lower. In contrast to the Arctic and Antarctica, where MP can accumulate due to a longer MP addition via snowfall [38], seasonal ice releases its trapped MP every year. By combining both processes, similar MP abundances in those regions are plausible.

In contrast to studies conducted in the Arctic [39,40,41] and Antarctica [42], the previous study conducted in the Baltic Sea did not find that MP abundances accumulated at higher concentrations in sea ice samples than the water surface [49]. However, in this study, we reached a different conclusion. A comparison of the reported MP abundances in sea ice by Geilfus et al. [49] (8–41 N L^−1^) and the results of the present study (22.3 ± 8.7 N L^−1^) reveals that MP abundances detected for the same region in surface waters (0.4 N L^−1^) [67] are at least one order of magnitude lower than those observed in sea ice. Higher MP concentrations observed by Zhou et al. [67] were detected in waters sampled in more southern regions or deeper water layers, with a maximum concentration of 27.7 N L^−1^. A subsequent study reported a maximum concentration of 3.3 N L^−1^ [68] for the Baltic Sea, but the majority of MP concentrations were lower (e.g., 2.2–2.7 [69]; 1.37 × 10^−3^ [70]; 9.2 × 10^−4^ [71]; max 8.2 × 10^−3^ N L^−1^ [72]).

In the comparison of MP concentrations detected in ice or in water samples, the method employed must also be considered. The results can vary significantly depending on the mesh size of the sampling net used, the sieves used during the separation processes of MP, or also during further digestion or density separation steps for MP determination [73]. For instance, Peeken et al. [37] considered particle sizes down to 11 μm, a size that was methodically infeasible to consider in the majority of sea ice studies (Table 2). In this size range alone, 67% of all identified MP particles were found. The majority of studies found a greater MP abundance with decreasing MP sizes in the marine system [37,38,74,75,76]. Consequently, it is also understandable that Kanhai et al. [39] found significantly lower MP concentrations in the similar area of the Arctic, as their considered size range of 100–5000 μm differs significantly from 11–5000 μm [37]. The use of plankton nets with a mesh size of 100–335 μm was preferred for the sampling of Baltic Sea water [70,71,72], which may result in the loss of significant quantities of smaller microplastics (MPs). Therefore, the most reliable comparison is a study with a similar MP size range as the present study and that of Geilfus et al. [49]. This was achieved by Zhou et al. [67], in which a 12-bottle rosette sampler system was used instead of plankton nets, and the resulting bulk water samples were filtered directly through a 5 μm filter. Due to the selective identification of MP, the lower detection limit of MP sizes is not known with certainty. However, it can be estimated to be below 100 μm. Stolte et al. [68] also employed a lower threshold of detection (55 μm), but their study was conducted in more southerly areas of the Baltic Sea, which may have resulted in higher concentrations [67].

## 5. Conclusions

The presence of MPs was confirmed in all five selected sea ice cores collected in March 2021. The MP abundances ranged from 13.7 to 37.1 N L^−1^, with the highest abundance observed at the northernmost station (119). Particle-shaped MPs were more frequently identified (64.9%) than fiber-shaped MPs (35.1%), although the particles were smaller in their size. The most frequently detected polymer type was PET, which is attributed to the fiber content, that provides dominantly PET (93.8%). For particulate-shaped MPs, PE (37.2%) was the major detected polymer type. Within this study, no correlation between MP abundances or masses and distances to the land, industries, rivers, or latitude was found, except PP masses, which negatively correlate with the distance to Luleå (Sweden). A negative correlation was also found for the same region and MP particle sizes, indicating that Luleå may be a potential source of larger MP particles.

Due to the highly dynamic nature of the ice layer within the Baltic Sea, including a multitude of rafting, ridging, freezing, and melting processes, no discernible trend of the vertical distribution was observed for MP throughout the ice core lengths. Instead, we found that the seasonal sea ice of the brackish Baltic Sea, which considerably differs from ice in saline waters, also exhibits MP accumulation compared to the surrounding waters. Moreover, the observed MP abundances were comparable to those reported in other sea ice studies conducted globally, including those in the Arctic and Antarctica.

The insights gained in this study offer valuable information regarding the distribution of microplastics (MPs) in brackish ice, emphasizing the necessity for further investigation into the mechanisms of MP accumulation in diverse marine environments. Further studies should concentrate on a more comprehensive examination of the physical processes that contribute to MP accumulation in ice, which will facilitate a more profound comprehension and more efficacious mitigation of MP pollution. The findings underscore the necessity for continuous monitoring and research to elucidate the intricate interactions between microplastics and marine ecosystems, with the ultimate objective of developing sustainable solutions to this global environmental issue.

## Figures and Tables

**Table 1:** Metadata of the sea ice core samples from the Gulf of Bothnia (Baltic Sea) for MP analysis.

Station	Date	Coordinates		Air Temp. (°C)	Ice Thickness (cm)	Water Salinity	Core Length (cm)
86	9 March 2021	64°22.730′ N	22°58.666′ E	−8.0	25	0.410	16
118	11 March 2021	65°02.051′ N	23°55.071′ E	−8.0	27	0.383	19
119	11 March 2021	65°03.578′ N	23°37.943′ E	−7.0	30	0.634	20
133	12 March 2021	64°40.369′ N	23°11.835′ E	−3.0	30	0.606	20
141	13 March 2021	64°28.132′ N	23°10.658′ E	−2.5	25	0.689	16

**Table 2:** Previous investigations of microplastic abundances in sea ice.

Location	Ice Type	MP Size Range (μm)	MP Abundance (N L^−1^)	Reference
Central Arctic	multi-year	<5000	0.038–0.234	[35]
Central Arctic	land-fast/pack	11–5000	1100–12,000	[37]
Western Arctic	multi-year	10–5000	23 ± 11	[41]
Central Arctic	first/multi-year	100–5000	2–17	[39]
Western Arctic	ice floes	20–5000	43.2 ± 32.0	[40]
Svalbard	seasonal	50–5000	158 ± 155	[45]
Antarctica	fast ice	11–5000	11.71	[42]
Novik Bay, Sea of Japan	first-year	25–5000	428	[44]
Gulf of Bothnia, Baltic Sea	seasonal	63–5000	8–41	[49]
Gulf of Bothnia, Baltic See	seasonal	10–5000	22.3 ± 8.6	this study

## Data Availability

All sample data, as well as MP abundances, masses, polymer types and sizes, are provided in the Supplementary Materials or are available from the authors upon request. Ice coverage data and updates are freely available at https://www.bsh.de/EN/DATA/Predictions/Ice_reports_and_ice_charts/ice_reports_and_ice_charts_node.html;jsessionid=44D2F4B7864B3A3A8646C64D0AE95709.live21302 (accessed on 20 July 2024 as well as at [66]).

## Supplementary Materials

The following supporting information can be downloaded at: https://www.mdpi.com/article/10.3390/toxics12080542/s1, Table S1: Distances of the sampling stations to adjacent land and potential microplastic sources, such as the big city Oulu, the iron and steel industry in Raahe and Luleå, and the River Kemi, which has a large catchment area; Table S2: Blank corrected MP numbers and masses per slice and ice core.
