# Supplementary material for: Microplastic Particles and Fibers in Seasonal Ice of the Northern Baltic Sea
Source: Toxics. 2024 Jul 26;12(8):542. doi: 10.3390/toxics12080542 (PMC11359375; doi:10.3390/toxics12080542)
Supplement: Supplementary file 1 [file toxics-12-00542-s001.zip › toxics-3097379-supplementary.pdf]

Table S1. Distances of the sampling stations to adjacent land and potential microplastic sources, such as the big city Oulu, the iron and steel industry in Raahe and Luleå, and the River Kemi, which has a large catchment area.

station	Oulu (km)	Swedish coast (km)	Finnish coast (km)	Raahe (km)	Kemi River (km)	Luleå (km)
86	134	66	33	75	170	137
118	70	58	29	47	86	87
119	83	55	43	58	88	100
133	110	78	47	57	135	110
141	121	74	33	62	157	130

Table S2. Blank corrected MP numbers and masses per slice and ice core.

station/slice	MP number (N)	MP mass (µg)	MP abundance (N L^{-1})	MP mass (µg L^{-1})
86_1	25	18.25±1.91	86.21	62.93±6.59
86_2	2	0.02±0.01	5.88	0.07±0.03
86_3	12	3.95±0.41	40.00	13.16±1.37
86_4	3	2.54±0.08	8.57	7.25±0.24
86_5	2	1.96±0.07	5.56	5.45±0.19
118_1	6	7.69±0.83	23.08	29.56±3.19
118_2	5	0.00±0.54	15.15	0.00±1.63
118_3	11	25.08±0.75	30.56	69.65±2.09
118_4	2	0.00±0.59	7.14	0.00±2.10
118_5	3	0.00±0.55	7.50	0.00±1.38
118_6	3	0.00±0.53	9.09	0.00±1.61
119_1	13	15.35±0.13	27.66	32.65±0.27
119_2	17	3.10±0.09	53.13	9.69±0.27
119_3	13	1.33±0.04	37.14	3.80±0.11
119_4	16	10.43±0.30	41.03	26.73±0.76
119_5	10	9.82±0.29	30.30	29.76±0.88
133_1	6	16.44±0.43	13.33	36.54±0.96
133_2	6	4.21±0.11	16.67	11.69±0.31
133_3	11	12.33±0.26	30.56	34.26±0.73
133_4	8	5.43±0.12	19.05	12.93±0.29
133_5	7	18.16±0.11	15.91	41.28±0.25
141_1	4	1.14±0.09	5.00	3.51±0.09
141_2	2	1.24±0.11	11.43	3.25±0.28
141_3	8	1.17±0.13	5.00	3.11±0.26
141_4	8	3.94±0.18	22.86	3.35±0.39
